# A Quality Improvement Project (QI) on Screening for Rapid Eye Movement Sleep Behaviour Disorder (RBD) in Patients Referred to Trafford Memory Assessment and Treatment Service (MATS), Part of Greater Manchester Mental Health Trust (GMMH)

**DOI:** 10.1192/bjo.2022.92

**Published:** 2022-06-20

**Authors:** Rachel Moir, Ruth Pye-Jones, Amit Sindhi, Boben Benjamin

**Affiliations:** Greater Manchester Mental Health, Manchester, United Kingdom

## Abstract

**Aims:**

Lewy Body Dementia (LBD) is predicted to be under-diagnosed in the general population. RBD is one of the four core clinical criteria for the diagnosis of LBD. Longitudinal studies of RBD show strong association with LBD, so there is potential for early identification of LBD and subsequent management. We aimed to screen 100% of patients referred to Trafford MATS for RBD.

**Methods:**

We performed three Plan-Do-Study-Act (PDSA) cycles; in the first cycle we introduced a validated RBD screening question, from the DIAMOND-Lewy study, to the initial memory assessment proforma. This asked ‘Have you ever been told that you “act out your dreams” while sleeping (punched or flailed arms in the air, shouted or screamed)?’

In the second PDSA cycle, we delivered a RBD and LBD educational package to the specialist memory nurses who undertake the initial assessments. In the third PDSA cycle reminders were sent to the team to use the new assessment proforma.

We collated data from patients who had undergone an initial memory assessment between 06/04/21- 22/06/21 from the trusts electronic database.

**Results:**

Initial baseline data showed that 0% of initial assessments screened for RBD; at the end of PDSA one this was 100% and 75% at the end of PDSA two. This increased to 100% at the end of the last PDSA cycle. The main reason for non-completion of the screening question was use of the old proforma.

4/152 patients screened positive; patients were diagnosed with Alzheimer's disease, delirium, vascular dementia and mixed Alzheimer‘s disease and vascular dementia, respectively.

**Conclusion:**

The introduction of a RBD screening question into the MATS initial assessment proforma improved screening for RBD. We think the variation in screening compliance rates was likely due to practitioners using old assessment proformas, hence sending reminders of the new proforma.

A limitation of the project was that some patients did not have a bed partner, which makes identification of the disorder more difficult.

Since the completion of the project, we have circulated a news bulletin through the Dementia United charity to raise awareness of our QI project nationally and also discussed the project with the Lewy Body society. Whilst our project has not yet identified a patient with LBD, we feel that introducing this screening question is a very easy and reproducible change to implement and RBD should be screened for in all memory patients.

